# Functional Activity of Recombinant Forms of Amh and Synergistic Action with Fsh in European Sea Bass Ovary

**DOI:** 10.3390/ijms221810092

**Published:** 2021-09-18

**Authors:** Cinta Zapater, Ana Rocha, Gregorio Molés, Alessia Mascoli, Soledad Ibañez, Silvia Zanuy, Ana Gómez

**Affiliations:** 1 Department of Fish Physiology and Biotechnology, Instituto de Acuicultura Torre de la Sal, Consejo Superior de Investigaciones Científicas (CSIC), Torre la Sal, 12595 Castellón, Spain; cinta.zapater@csic.es (C.Z.); anasanrocha@gmail.com (A.R.); gregorio.moles@inia.es (G.M.); alessia.mascoli@iats.csic.es (A.M.); sole@iats.csic.es (S.I.); silviazanuydoste@gmail.com (S.Z.); 2 Centro Interdisciplinar de Investigação Marinha e Ambiental (CIIMAR), Terminal de Cruzeiros do Porto de Leixões, 4450-208 Matosinhos, Portugal

**Keywords:** anti-Müllerian hormone, fish reproduction, ovary, granulosa cells, theca cells, gonadotropins, steroid hormones

## Abstract

Although anti-Müllerian hormone (AMH) has classically been correlated with the regression of Müllerian ducts in male mammals, involvement of this growth factor in other reproductive processes only recently come to light. Teleost is the only gnathostomes that lack Müllerian ducts despite having *amh* orthologous genes. In adult teleost gonads, Amh exerts a role in the early stages of germ cell development in both males and females. Mechanisms involving the interaction of Amh with gonadotropin- and growth factor-induced functions have been proposed, but our overall knowledge regarding Amh function in fish gonads remains modest. In this study, we report on Amh actions in the European sea bass ovary. Amh and type 2 Amh receptor (Amhr2) are present in granulosa and theca cells of both early and late-vitellogenic follicles and cannot be detected in previtellogenic ovaries. Using the *Pichia pastoris* system a recombinant sea bass Amh has been produced that is endogenously processed to generate a 12–15 kDa bioactive mature protein. Contrary to previous evidence in lower vertebrates, in explants of previtellogenic sea bass ovaries, mature Amh has a synergistic effect on steroidogenesis induced by the follicle-stimulating hormone (Fsh), increasing E2 and *cyp19a1a* levels.

## 1. Introduction

Anti-Müllerian hormone (AMH), also known as Müllerian inhibiting substance (MIS), promotes the regression of the Müllerian duct during normal male sexual differentiation [1]. AMH belongs to the transforming growth factor β (TGF-β) superfamily and, as in all the members of this family, is translated as a precursor protein that must undergo cleavage to generate a mature protein (Amh_C_) [2]. AMH signals through a heterodimeric receptor complex consisting of two related serine/threonine kinase receptors, type 2 and type 1. Type 2 Amh receptor (AMHR2) is expressed in fetal and adult gonads of both sexes [3], in the brain [4], and in other tissues [5,6]. No specific AMH type 1 receptor has been identified to date, although it has been demonstrated that BMP proteins share type 1 receptors with AMH [7,8]. In mammals, circulating AMH is subject to complex regulation during the life cycle in a sexually dimorphic manner [9], and AMHR2 is also differentially expressed during gonadal development [3,10,11,12]. In recent years, it has become apparent that AMH has multiple effects in gonadal steroidogenesis and follicular development, in addition to its effect on Müllerian duct regression [13,14]. AMH blocks the differentiation of somatic precursor cells into mature Leydig cells, inhibits the ability of cAMP and FSH to induce the expression of steroidogenic enzymes such as aromatase [15,16,17], and plays an important role during folliculogenesis [18].

Although no structure similar to Müllerian ducts exists in teleosts, the existence of an *amh* orthologous gene has been described in several species, including Japanese eel [19], zebrafish [20], European sea bass [21], and medaka [22] among others [23]. Most teleosts present a male-biased *amh* expression during sex differentiation or, at least, in differentiated juvenile gonads [24,25,26]. In addition, Amh signaling regulates the proliferation of self-renewing type I germ cells during gonad development in medaka, as demonstrated by the hyperproliferation of these germ cells in the Amhr2/*hotei* loss-of-function mutant [27,28]. However, the expression of *amh* and localization of Amh polypeptide have been observed in Sertoli cells of the adult testis [19,20,22,24,29,30,31,32] and granulosa cells of previtellogenic and vitellogenic follicles in adult ovaries [20,22,29], suggesting that in teleosts Amh is involved in gonadal steroidogenesis and follicular development, as in mammals. Most existing studies concerning the physiological actions of Amh in adult teleosts have been conducted in males. They show that the role of Amh in teleost males is similar to that observed in mammals, preventing androgen-stimulated spermatogenesis [19,32]. On the other hand, it has been suggested that Amh is required for androgen synthesis and therefore, associated with steroidogenesis onset [33]. In adult female teleosts, there is a lack of information about the mechanisms of action of Amh. However, analysis of female medaka homozygous for the *hotei* mutation showed hypertrophic ovaries due to the uncontrolled proliferation of germ cells, and that follicular development is arrested at an early vitellogenic stage, suggesting that Amh is involved in vitellogenin uptake [27]. Recently, these results have been confirmed using zebrafish and Nile tilapia Amh/Amhr2 mutants, which showed the accumulation of previtellogenic follicles in hypertrophic and sterile ovaries [34,35,36]. In zebrafish, Amh probably plays a dual role in controlling folliculogenesis, by involving Fsh-Fshr signaling: limiting the formation and recruitment of primary growth (PG) follicles and promoting their transition to more advanced stages of secondary growth (SG) [36]. However, the exact mechanism by which Amh controls PG follicle recruitment and follicle transition from the PG to the SG phase remains largely unknown.

In the European sea bass (*Dicentrarchus labrax*), the *amh* gene has been isolated and its expression and that of alternatively spliced isoforms have been analyzed [21]. Recently, an *amhr2* gene was also isolated and used to study the activation and intracellular signaling pathway of a recombinant sea bass Amh produced in the Chinese hamster ovary (CHO) cell line. The gonad temporal expression profile of both ligand and receptor genes were evaluated and immunological detection was used to investigate the cellular localization of Amh in sea bass testis and to characterize native and recombinant Amh proteins [30]. In the present work, we report the production, cleavage, and secretion of a mature and active recombinant sea bass Amh in the *Pichia pastoris* system. The activity of the purified recombinant sea bass Amh and human Amh was studied in the sea bass Amhr2 receptor and in ovary cultures, and whether Amh modulates steroidogenesis in adult sea bass ovaries was tested by quantifying steroid production and *cyp19a1a* gene expression. In addition, we investigated the cellular localization of Amh and Amhr2 in sea bass adult ovaries. The results here presented to suggest a role for Amh during early vitellogenesis, involving the regulation of local ovarian steroidogenesis and an additive increase in the subsequent endocrine effect of Fsh during vitellogenesis.

## 2. Results

### 2.1. Production of Recombinant Sea Bass Amh in the Yeast P. pastoris

**Figure 1:**
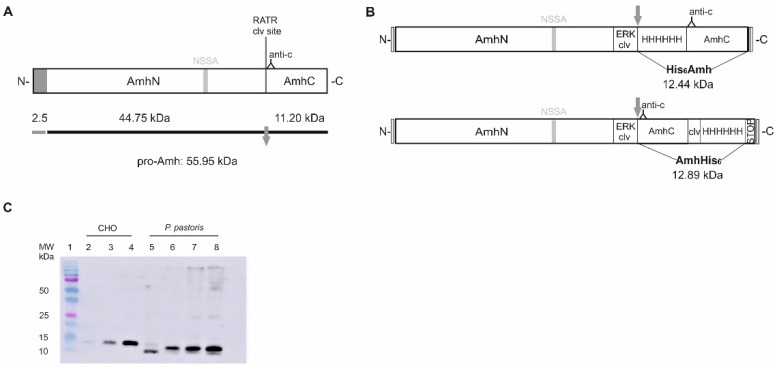
Sea bass anti-Müllerian hormone (Amh) proteins. (**A**) Structure of sea bass Amh preproprotein: Arg^426^-Ala-Thr-Arg (RATR) is the presumptive proprotein convertase cleavage site in the endogenous sea bass Amh. In agreement with the prediction results, the first 22 residues (2.5 kDa) correspond to the signal peptide (dark grey rectangle) and the Asn^321^ in NSSA sequon is *N*-glycosylated. After signal peptide cleavage, pro-Amh has a MW of 55.95 kDa. Proteolytic processing of pro-Amh yields 44.75 kDa Amh_N_ and 11.20 kDa Amh_C_ domains. The position of the C-terminal peptide used for antibody production is illustrated (anti-c); (**B**) Engineered sea bass His_6_Amh and AmhHis_6_: sea bass Amh signal peptide was removed to clone the mature gene in frame and downstream of the α-factor signal sequence in the pPIC9K plasmid. The putative protease cleavage site was changed to an EKR site (ERK clv) to allow cleavage of the sea bass Amh proprotein by *P. pastoris* enzyme Kex2p. A His_6_-tag (HHHHHH) was placed before (pPICK9-His_6_Amh) or after (pPICK9-AmhHis_6_) the mature peptide to facilitate the purification. AmhHis_6_ also includes an IEGR site (clv), placed *N*-terminal of the His_6_-tag, for the removal of the affinity tag by the Factor Xa protease; (**C**) Recombinant sea bass Amh proteins: Known concentrations (1, 2 and 4 ng/µL) of sea bass Amh produced in CHO cells [30] (lanes 2–4) were used to infer the concentration of purified sea bass His_6_Amh (lane 5, 1 µL) and AmhHis_6_ (lanes 6–8, 1, 3 and 4 µL) proteins produced in *P. pastoris*. A calibrated protein standard (in kDa) was used to estimate protein molecular weight (MW).

In an attempt to produce a cost-effective bioactive recombinant sea bass Amh, we engineered two vectors to be expressed in the yeast *P. pastoris*. In both constructs, the putative cleavage of the native hormone (R^426^ATR) site (Figure 1A and Figure S1) was changed to Glu-Lys-Arg for cleavage by yeast Kex2p enzyme, the yeast homolog of furin. Those two vectors differ in the position of a His_6_-tag, which was introduced to allow purification of the mature protein, and generate a His_6_Amh or an AmhHis_6_ protein. The essential elements of the constructs are shown in Figure 1B.

For each construct, the amount of recombinant protein produced was screened by Western blot in cell extracts and up-concentrated media of several clones, using the highest expressing clone in all subsequent experiments. High levels of recombinant sea bass Amh were produced, and no proteolytic treatment was necessary to obtain the mature protein. The yeast Kex2 protease cleaved the pro-peptide in vivo, generating a mature sea bass Amh_C_ of 12–15 kDa, which was secreted into the culture media, as detected by Western blot after purification (Figure 1C). The sea bass His_6_Amh and AmhHis_6_ proteins were slightly smaller than that previously obtained using CHO cells [30] and their relative sizes also differed between both (Figure 1C). Additionally, little difference in expression levels was observed between pPICK9-His_6_Amh and pPICK9-AmhHis_6_ constructs, indicating that the position of the His_6_-tag had little effect on the expression levels of sea bass Amh (data not shown).

### 2.2. Functional Characterization of Sea Bass Amh

**Figure 2:**
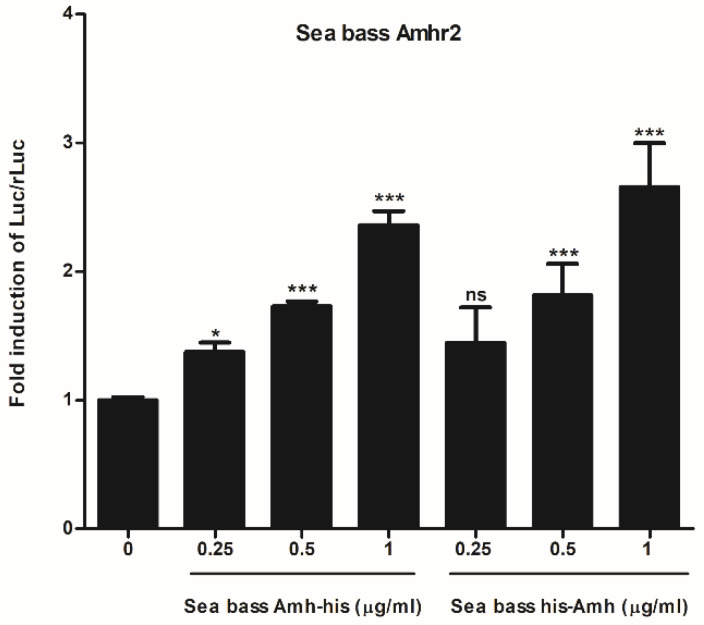
Recombinant sea bass AmhHis_6_ and His_6_Amh induce sea bass type 2 Amh receptor (Amhr2)-dependent BRE-luc reporter activity. African green monkey kidney fibroblast-like (COS-7) cells were transiently transfected with sea bass Amhr2, along with the Bre-Luc reporter and pRL-TK plasmids. The cells were incubated with three different concentrations of purified recombinant sea bass AmhHis_6_ or His_6_Amh for 24 h in a 1% FBS culture medium. Firefly luciferase activities were normalized to pRL-TK *Renilla* luciferase. The results shown are the fold induction of normalized luciferase activity, expressed as relative light units (RLUs), of each treatment over the values of cells treated with culture media obtained from *P. pastoris* expressing the empty pPIC9K vector. Data represent the mean ± SEM of three independent experiments (in triplicate for each condition) and were analyzed by ANOVA followed by Tukey’s significant interaction test. Bars with asterisks are significantly different (* *p* < 0.05; *** *p* < 0.001) and ns indicates there were no significant differences.

**Figure 3:**
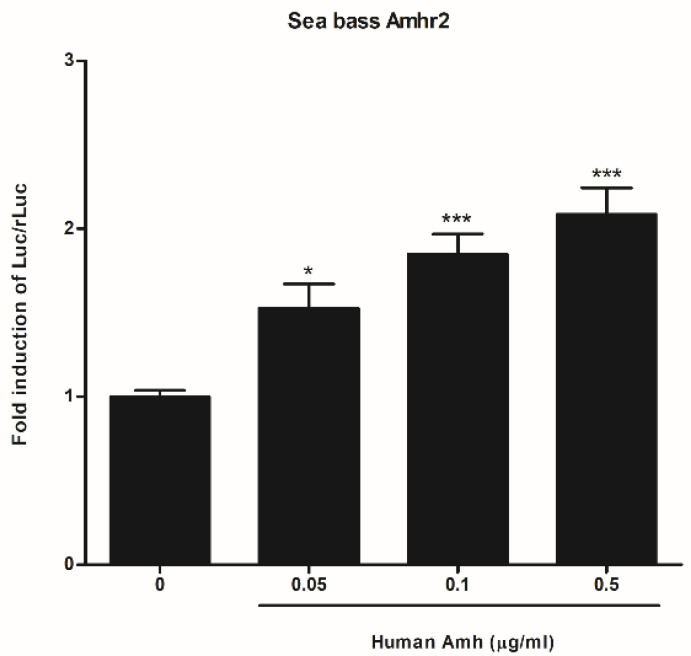
Human AMH induces sea bass Amhr2-dependent BRE-luc reporter activity. COS-7 cells were transiently transfected with sea bass Amhr2, along with the BRE-Luc reporter and pRL-TK plasmids. The cells were incubated with three different concentrations of hAMH for 24 h in a 1% FBS culture medium. Firefly luciferase activities were normalized to pRL-TK *Renilla* luciferase. The results shown are the fold induction of corrected luciferase activity (expressed as RLUs) over the values of control cells treated with the hAMH diluent BSA-HCl in a 1% FBS culture medium. Data represent the mean ± SEM of three independent experiments, carried out in triplicate for each condition, and were analyzed by ANOVA followed by Tukey’s significant interaction test. Bars with asterisks are significantly different (* *p* < 0.05; *** *p* < 0.001).

To test the bioactivity of recombinant sea bass His_6_Amh and AmhHis_6_ proteins, we used a cell-based reporter assay that depends on sea bass Amhr2 activity [30]. Both recombinant sea bass Amh were able to increase the activity of the BRE-Luc reporter gene in a dose-dependent manner (Figure 2). Considering the slightly higher production scale of AmhHis_6_ with respect to His_6_Amh, only the former was used in the explant culture experiments. In a previous study we demonstrated the capacity of sea bass Amh to activate human AMHR2 [30], and, to complete this interspecies functional comparison, the ability of human AMH to activate the sea bass Amhr2 was tested. The results showed significantly higher luciferase activity than that obtained with the control at all tested doses, and a dose-response trend, as observed for AmhHis_6_ (Figure 3).

### 2.3. Immunolocalization of sbsAmh and sbsAmhr2 in Sea Bass Ovary

**Figure 4:**
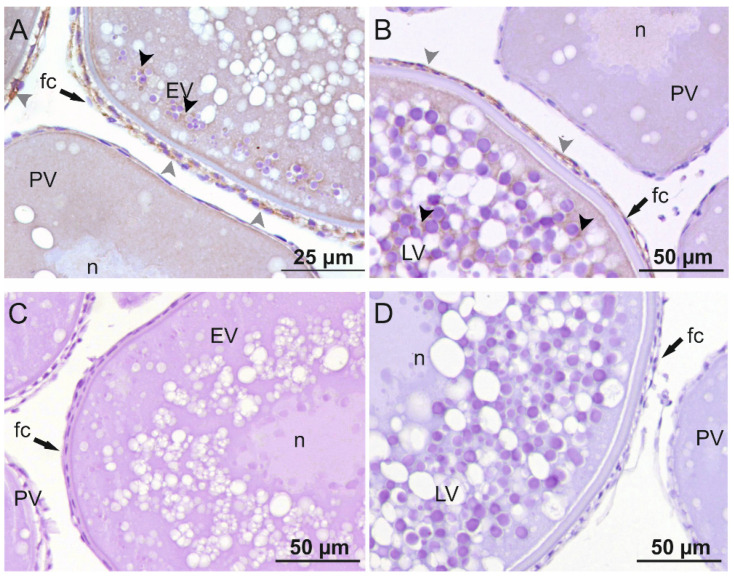
Immunolocalization of Amh in sea bass ovaries at different developmental stages. Photomicrographs of (**A**) early vitellogenic (EV) and (**B**) late-vitellogenic (LV) follicles showing Amh staining signal in follicular cells (gray arrowheads) and the cytoplasm (black arrowheads); (**A**,**B**) The signal is absent in previtellogenic follicles (PV); (**C**) Control sections of early vitellogenic and (**D**) late-vitellogenic follicles without primary antibody. n: nucleus; fc: follicular cell.

When the immunodetection of Amh in sea bass ovaries was carried out at different stages of development (Figure 4), Amh could not be detected in the follicular cells or in the oocyte in previtellogenic ovaries. During early and late-vitellogenesis, Amh was observed in the follicular cells surrounding the oocyte, in both theca and granulosa cells. In addition, a faint signal could be noticed inside the oocyte surrounding yolk granules in both vitellogenic stages (Figure 4A,B).

**Figure 5:**
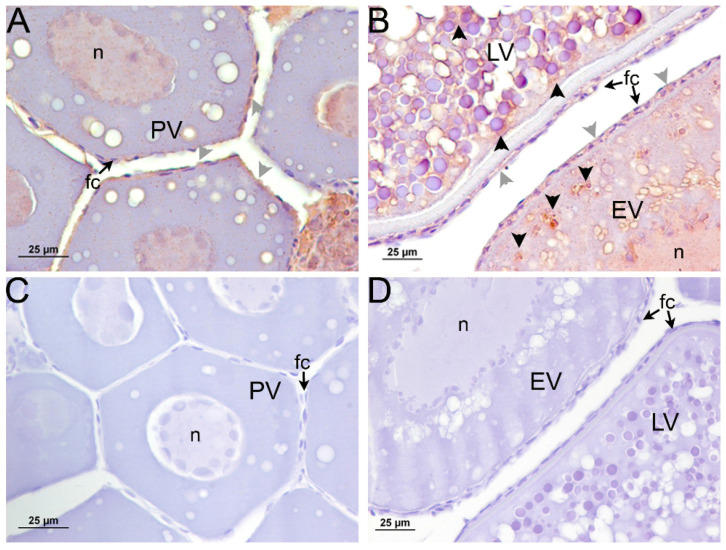
Immunolocalization of Amhr2 in sea bass ovaries at different developmental stages. Photomicrographs of follicles in (**A**) previtellogenesis (PV); (**B**) early vitellogenesis (EV) and (**B**) late-vitellogenesis (LV), showing an Amhr2 staining signal mainly in follicular cells (gray arrowheads) but also surrounding yolk globules inside the oocyte (black arrowheads); Control sections of (**C**) previtellogenic and (**D**) early-, late-vitellogenic follicles without primary antibody. n: nucleus; fc: follicular cell.

Western blot analysis of positive control protein lysates from cells expressing sea bass Amhr2, previtellogenic ovaries, and follicular cells using the new rabbit anti- sea bass Amhr2 antibody revealed a predominant band of ≈53 kDa in accordance with the theoretical molecular weight of sea bass Amhr2 (Figure S2). Using this validated antibody, we were able to detect the Amhr2 (Figure 5) in the follicular cells surrounding the oocyte, inside the oocyte surrounding yolk granules, and in the nucleus in both previtellogenic and vitellogenic ovaries (Figure 5A,B,D). A secondary antibody control showed that the label was specific to both primary antibodies (Figure 4C,D and Figure 5C,D).

### 2.4. Expression Pattern of amh and amhr2 in Follicular Cells

**Figure 6:**
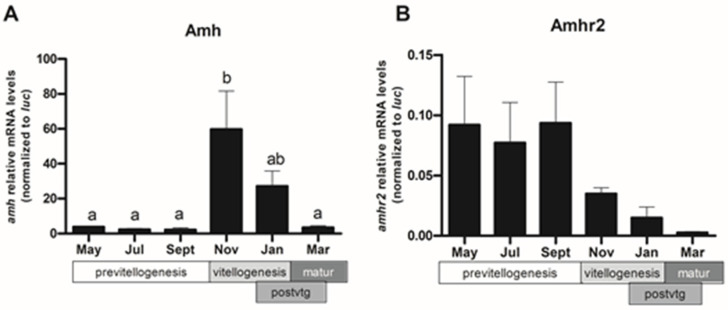
Relative expression of *amh* (**A**) and *amhr2* (**B**) in sea bass ovarian follicular cells during the reproductive cycle. Values represent the mean ± SEM (*n* = 3–4 fish/month) of each month and were analyzed by ANOVA followed by Tukey’s significant interaction test. Different significance levels (*p* < 0.05) are indicated with different letters above the bars, except for *amhr2* (*p* = 0.1824).

After localizing Amh and Amhr2 in the follicular cells, changes of *amh* and *amhr2* expression were determined by RT-qPCR in follicular cells isolated from sea bass ovaries at different stages of maturation (Figure 6). The levels of *amh* mRNA were low from May to September during previtellogenesis, then dramatically increased in November, when vitellogenesis started and decreased again during post-vitellogenesis (postvtg) to reach low levels in March, during the spawning period (matur) (Figure 6A). There were no significant changes in *amhr2* expression at any time during the reproductive cycle, although the expression pattern was the inverse of that of the *amh*. The highest expression levels occurred during previtellogenesis and dropped when vitellogenesis started (Figure 6B).

### 2.5. Synergistic Effect of Amh on Fsh-Induced Steroidogenesis in Previtellogeneic Ovaries

**Figure 7:**
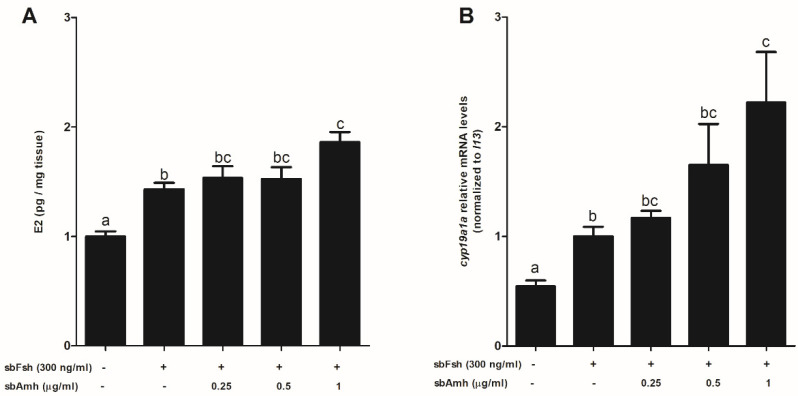
In vitro effect of sea bass Amh and sea bass Fsh on (**A**) E2 levels and (**B**) *cyp19a1a* expression in previtellogenic/early-vitellogenic sea bass ovarian explants. Data are shown as the means ± SEM and correspond to four separate experiments (*n* = 4) with 6 different replicates each and were analyzed by ANOVA followed by Tukey’s significant interaction test. Statistical differences (*p* < 0.05) are indicated with different letters above the bars.

**Figure 8:**
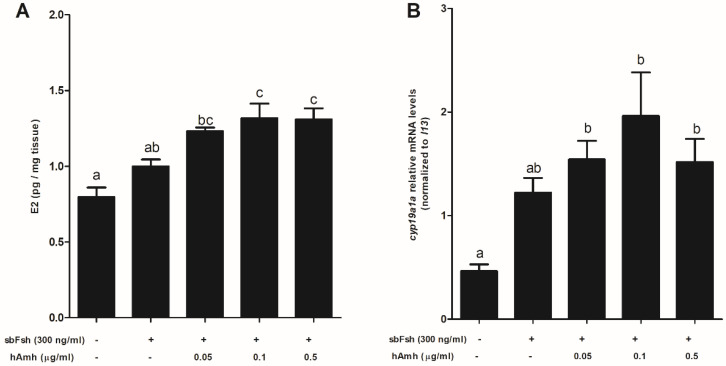
In vitro effect of human AMH and sea bass Fsh on (**A**) E2 levels and (**B**) *cyp19a1a* expression in previtellogenic/early vitellogenic ovarian explants. Data are shown as the means ± SEM and correspond to four separate experiments (*n* = 4) with 6 different replicates each and were analyzed by ANOVA followed by Tukey’s significant interaction test. Statistical differences (*p* < 0.05) are indicated with different letters above the bars.

To assess the activity of Amh in adult sea bass ovaries, explant cultures of the ovaries were treated with 300 ng/mL of Fsh alone or in combination with different doses of purified sea bass Amh_C_ (Figure 7) and human AMH (Figure 8). The effect of externally added hormones became more evident when their endogenous levels in the target tissue were basal. The lowest expression levels of *amh* were observed in previtellogenesis (Figure 6A; [30]). In addition, the highest values of *amhr2* expression were seen during that developmental stage ensuring a response to the exogenously added hormone (Figure 6B; [30]). For this reason, we used previtellogenic ovaries with already visible cortical alveoli. Levels of E2 in culture media and *cyp19a1a* tissue expression were measured using specific EIA and qPCR, respectively (Figure 7). The results show that E2 levels increased in response to Fsh treatment. The addition of sea bass Amh_C_ resulted in higher E2 production than obtained with Fsh alone. This increase in E2 production was significantly different for the highest dose of Amh, pointing to a synergistic effect of Amh on Fsh-induced E2 synthesis in previtellogenic ovaries (Figure 7A). The results for *cyp19a1a* expression showed a similar profile to that observed for E2 production. A significant increase in *cyp19a1a* transcript levels was observed in Fsh-treated ovaries. When combined with sea bass Amh treatment, this increase was significantly different from that of Fsh and behaved in a dose-responsive manner (Figure 7B).

Regarding human AMH, the results were similar to those observed for sea bass Amh-induced E2 production (Figure 8A) but they differed slightly in the case of *cyp19a1a* expression, where all the tested doses of human AMH produced the same significant increase when combined with Fsh (Figure 8B).

## 3. Discussion

This work focuses on Amh function in the sea bass ovary. Using a recombinant sea bass Amh produced in the methylotrophic yeast *Pichia pastoris*, we found that contrary to previous reports concerning the zebrafish model organism, Amh has an additive effect on Fsh-stimulated steroidogenesis, increasing *cyp19a1a* expression and estrogen production in adult previtellogenic ovaries cultured in vitro. These results were in line with the cellular localization of both sea bass Amh and its specific receptor, the Amhr2, in ovaries at different stages of gonad development.

We previously produced a bioactive recombinant sea bass Amh using CHO cells [30], which was engineered to contain changes from the native sea bass sequence in order to increase endogenous cleavage by the protein convertases present in CHO cells and a His-tag to facilitate its purification. However, cleaved Amh only represented ≈ 5% of the total protein secreted into the culture media, creating the need for subsequent in vitro cleavage with plasmin. Overall, the cost and effort of production were high, and expression low compared with that achievable in microbial systems. In order to overcome these limitations, the present work aimed to expressed sea bass Amh in the methylotrophic yeast *P. pastoris*. In previous reports in the literature on the overexpression of mammalian TGF-β proteins in yeast systems [37,38,39], high yields of mature protein were only obtained when the protease cleavage sites between the pro-domain and the mature signaling dimer were altered to more closely match the cleavage sites of endogenous proteases in yeast. Therefore, we mutated the putative monobasic cleavage site Arg^426^-Ala-Thr-Arg to a Glu-Lys-Arg site for cleavage by the Kex2p enzyme, the yeast homolog of mammalian serine proteases, allowing secretion of the mature Amh to be readily purified from the culture supernatants via IMAC. In this way, the use of *P. pastoris* as a host for the expression of recombinant sea bass Amh solved the technical constrain of incomplete/absent processing observed in mammalian cell lines. We have also learned from previously published studies that the position of the purification tag may affect the bioactivity of recombinant proteins from the TGF-β family [40]. Therefore, we engineered two vectors that differ in the position of the His_6_-tag. Our data indicate that the position of the tag affected neither the expression levels nor the proteolytic cleavage of sea bass Amh recombinant proteins and did not interfere with their bioactivity. Both *P. pastoris* recombinant proteins activate sea bass Amhr2 with a similar fold increase in luciferase activity over the control. These results match those for sea bass Amh_C_ produced in CHO cells. Accordingly, the mature ligand of human AMH can host an “N-terminal” FLAG-tag while maintaining bioactivity [41].

The production of recombinant Amh proteins has only been achieved in two other teleosts: black porgy [42] and zebrafish [32]. For the first-mentioned species, no Tag was added, while in zebrafish a bioactive recombinant Amh was produced in human cells with a His-tag introduced after Pro^33^ (GenBank accession number: AY721604) in the N-terminus of pro-Amh to allow its purification from culture media before treatment with plasmin. The resulting protein is therefore a mix of the N-terminal pro-region and untagged mature domain. A hypothetical effect of the position of the affinity purification tag on the bioactivity of mature zebrafish Amh cannot, therefore, be deduced and may even make it more difficult to understand, considering the impossibility of identifying the gene encoding a zebrafish Amh receptor [34]. The sea bass Amh proteins produced by *P. pastoris* have a slightly lower size than that previously obtained using CHO cells. These size differences can be explained to some extent by differences in post-translational modifications between the CHO and *P. pastoris* expression systems. Yeast micro-organisms are capable of performing typical eukaryotic post-translational modifications. However, and in contrast to *N*-glycosylation in mammalian cells, yeast performs hypermannosylation and lacks the ability to generate sialylated *N*-glycans (reviewed in [43]). According to the NetNGlyc server, there are four potential N-linked glycosylation sites in the amino acid sequence of sea bass Amh. However, all of them are in the N-terminal region so they could not account for the size differences in the sea bass Amh_C_ [30]. While zebrafish Amh is *N*- and possibly also *O*-glycosylated in human cultured cells, glycosylation taking place in both N- and C-terminal halves, the endogenous protein present in zebrafish testis is not. Glycosylation is not only species- and cell-specific but is also affected by culture conditions, producing differences between recombinant glycoproteins and their endogenous counterparts and also impacting the reproducibility of production processes [44]. Therefore, even though we did not perform glycosylation analysis studies, it is plausible to conclude that the observed differences in the size of sea bass recombinant proteins are due to specific post-translational modifications that can be attributed to the expression system used. The differences in size between both *P. pastoris* secreted recombinant Amh proteins could be a consequence of the presence in the AmhHis_6_ of four extra amino acids corresponding to the Ile-Glu-Gly-Arg cleavage site added for the eventual removal of the affinity tag by the Factor Xa protease.

The expression sites of *amh* and localization of Amh protein in the ovary have been widely described in mammals and are well conserved among different species [10,45,46]. The expression of *amh* and immunoreactivity are influenced both by the degree of follicular development and by the age of the animal. In this work, we show that in sea bass, *amh* expression in the follicular cells is highest during vitellogenesis. Accordingly, during vitellogenesis, Amh protein can be detected in the follicular cells, as well as in the germ cells surrounding yolk globules. Most studies have found that Amh is located exclusively in granulosa cells, although two studies in humans [47] and goats [48] showed AMH immunostaining in oocytes (all follicular stages) and primordial follicles. During postnatal development in mammals, the signal is practically null in primordial follicles and is first observed in young growing follicles. The strongest signal appears in preantral and small antral follicles and disappears rapidly with increasing follicle size. Staining is least intense during ovulation or at follicular atresia [46,49]. The localization of AMH in mammals agrees with its well-known role as an inhibitor of the activation of primordial follicles [18], although in one study involving in vitro experiments it was observed that AMH increased the proportion of growing follicles [50], suggesting that AMH could activate folliculogenesis and steroidogenesis, in line with that observed in this work with sea bass. In teleosts, on the other hand, the sites of *amh* expression in the ovary vary greatly among species, and the localization of Amh protein by immunostaining has been described only in black porgy [42], orange-spotted grouper [51], Nile tilapia [35] and, in the present study, in sea bass. As observed in mammals, there is some controversy on the expression pattern of *amh* in germ cells, with reports on the presence of transcripts inside the oocytes of growing follicles in zebrafish ovaries [52]. In our previous work in sea bass [30], *amh* expression levels in the whole ovary increased during vitellogenesis and were maximum in maturation and ovulation. However, when analyzing in the present study *amh* expression in isolated follicular cells, *amh* expression decreased in maturation, after the vitellogenesis stage, meaning that the high expression levels previously reported during maturation-ovulation would correspond to mRNA molecules present inside the oocyte.

Even though *amh* expression may not be restricted to the somatic cells in fish, follicular cells seem to be the main source of *amh* expression in the ovaries of most fish species, as observed in other vertebrates. Studies using in situ hybridization in zebrafish and medaka show that *amh* is expressed in granulosa cells of previtellogenic and vitellogenic oocytes [20,23]. In the hybrid fish *Squalius alburnoides*, *amh* is also expressed in the adult ovaries, more specifically in the follicular cells surrounding the primordial and primary oocytes [29]. By contrast, in Japanese flounder *amh* is male-specific during both sex differentiation and adult life, and no expression is observed in the ovary [24]. Although *amh* is already expressed in primary and previtellogenic oocytes, the presence of Amh protein in the ovary is not detected until the entry of vitellogenesis, as observed in black porgy [42], orange-spotted grouper [51], and in this work, with the exception of Nile tilapia, in which it is found in the primary growth stage but not during vitellogenesis [35]. Localization of Amh in the adult ovary indicates that this growth factor has an important role during ovarian development, as observed in mammals, although its specific functions are still not known and, probably, may vary depending on the species.

In rodents and humans, *AMHR2* colocalizes with AMH, mainly in the granulosa cells of preantral and small antral follicles, with almost no expression in large antral follicles and corpus luteum [3,10]. However, *AMHR2* expression was also observed in theca cells of preantral and small antral follicles in adult rats and human ovaries, but, unlike the granulosa cell expression, theca cells continue to express *AMHR2* in antral and early atretic follicles and the corpus luteum [53,54,55]. Localization of the *AMHR2* in granulosa cells of early antral follicles and theca cells of more advanced follicles suggests an autocrine and paracrine role in ovarian steroidogenesis that differs from its role as an inhibitor of folliculogenesis. In this study, Amhr2 was localized in the follicular cells in previtellogenic and vitellogenic oocytes but also in germ cells in all stages up to late vitellogenesis. In female teleost, *amhr2* transcripts and Amhr2 protein have been localized in the follicular cells surrounding vitellogenic oocytes [22,52,56], with the exception of Nile tilapia, where Amhr2 was localized in germ cells and follicular cells of stage I ovaries [35].

In the gonads of adult fish, Amh seems to play a role during the early stages of germ cell development in both males and females [23], although most of the data in this respect comes from male fish. These include experiments performed in the Japanese eel [19], zebrafish [32,33,57] and medaka [27,28]. It is important to emphasize that in zebrafish, the species in which most studies have been performed, the identity of the gene encoding an Amh receptor is still unknown. Therefore, even though Amh has a function in zebrafish gonad physiology, putative target cells and intracellular pathways activated by Amh remain to be discovered. To date, the available information regarding Amh actions in fish ovaries is limited to reverse genetic studies performed in two model fish species, medaka and zebrafish, and in Nile tilapia. The absence of Amh signaling in these species results in ovaries composed mostly of perinucleolar oocytes, which are arrested in the early vitellogenesis stage [27,33,34,35,36]. It seems that in female teleosts Amh has a role in the development of the gonad, including the sex-dependent regulation of germ cell proliferation and folliculogenesis [27]. We have previously shown that the activation of Amhr2 by Amh triggers Smad-dependent downstream signaling in the European sea bass ([30]; also in this work). In the present study, we show, for the first time in a lower vertebrate, the direct in vitro effects of Amh administration on previtellogenic ovaries. The results point to an additive increase in Fsh-induced *cyp19a1a* expression and E2 release in those ovarian explants treated with Amh and suggest a role for Amh in ovarian steroidogenesis. In other teleost species, it is not clear whether Amh can affect *cyp19a1a* expression or not. In zebrafish [20] and Patagonian pejerrey [58], the expression pattern of *amh* is contrary to that of aromatase. In medaka [22], *amh* and *cyp19a1a* expression are independent of each other and, furthermore, medaka *hotei* mutants show no up-regulation of *cyp19a1* [27]. By contrast, in Atlantic cod [59], the ovarian expression of *cyp19a1a* and *amh* increased concomitantly with increasing plasma estradiol levels during vitellogenesis, which agrees with the results obtained in coho salmon [60], where *amh* expression starts to increase, along with *fshr* expression, just before vitellogenesis. Furthermore, recent studies in zebrafish [34] and Nile tilapia [35] show that *cyp19a1a* levels were significantly downregulated in the ovaries of Amh mutant fish. In the European sea bass, *cyp19a1a*, *fshr* and E2 levels begin to increase during early vitellogenesis, coinciding with an increase in *amh* expression in the ovary and in follicular cells ([30,61] and this work). These observations agree with the results obtained in the in vitro experiments reported in the present work. Fsh levels peak during early-vitellogenesis in European sea bass [62] and induce E2 production, as observed using in vitro ovarian cultures [63]. In addition, *amhr2* is already expressed during previtellogenesis, suggesting that the Amh-signaling machinery is ready before vitellogenesis onset, and the intracellular signaling set in motion by this growth factor could be serving as a complement to gonadotropin-stimulated E2 production during vitellogenesis.

A promoting role of Amh in steroidogenesis is in contrast to the results obtained in mammals. Experiments using AMH knockout mice suggest an involvement of AMH in mouse primordial follicle selection and growing follicle cyclic recruitment, attenuating the effect of FSH on follicle development [64,65], while experiments using human granulosa lutein cell cultures showed that AMH significantly inhibited FSH-induced E2 production, along with a concomitant reduction in aromatase mRNA and protein levels [66]. However, a recent study in Amh-knockout zebrafish proposes dual roles for Amh in fish gametogenesis that could reconcile these contradictory results [36]. On the one hand, the authors of this study propose that Amh inhibits the formation of new follicles, which is in line with findings in mammals and in other fish species, as described above, resulting in the accumulation of previtellogenic follicles in mutants lacking Amh signaling [27,28,35,36]. However, on the other hand, the Amh-deficient zebrafish mutant also reveals a new paracrine role for Amh in promoting the transition of previtellogenic follicles from primary to secondary growth, which probably acts in synergy with pituitary Fsh signaling [36]. This advancement towards vitellogenesis entry would be in line with our present results on Amh action in ovarian explants of sea bass.

## 4. Materials and Methods

### 4.1. Animal and Tissue Sampling

European sea bass fish were maintained at the Instituto de Acuicultura Torre de la Sal facilities (Castellón, Spain, 40° N) under natural photoperiod and temperature conditions before being anesthetized with an overdose of ethyl 3-aminobenzoate methanesulfonate (MS-222; 300–400 mg/L; Sigma-Aldrich^®^, St. Louis, MO, USA) and euthanized by decapitation. Ovaries (*n* = 18) in different maturation stages were sampled from adult females (2.666 ± 811 g) during a complete reproductive year, and follicular cells were isolated, as previously described [67]. The connective tissue capsule was removed, and the tissue was transferred to Medium 199 (Gibco, Life Technologies™ Ltd., Paisley, Scotland, UK) diluted 4:1 with distilled water. Follicle cells were separated by trypsinization at 37 °C. Trypsin activity was inhibited with 10% fetal bovine serum (FBS) and supernatant fractions (containing the follicular cells) filtered through a 100 µm pore size filter. For in vitro tissue culture, ovaries (*n* = 6) from adult females (2.636 ± 1249 g) were collected during October. For each animal, the stages of ovarian development were determined by histological analysis following previously established criteria [68].

### 4.2. Expression Plasmids

The expression plasmids pPICK9-His_6_Amh and pPICK9-AmhHis_6_, which contain modified sea bass *amh* cDNAs, were generated in several steps. First, an *amh* cDNA fragment coding for the N-terminal domain of sea bass Amh (GenBank accession number: AM232701.1) was PCR amplified with primer pair BamHIamh1–amh2 (primer details provided in Table S1) using cDNA from testes of juvenile sea bass as a template. The cDNA fragment corresponding to the C-terminal region of sea bass Amh was PCR amplified with primers amh3–amh4EcoRI. For this reaction, a plasmid containing a fragment of sea bass *amh*, previously isolated by testis cDNA library screening [21] was used as a template. Equal quantities of each fragment were joined in an overlapping PCR reaction with primers BamHIamh1–amh4EcoRI. The resulting PCR product, consisting of the complete *amh* ORF, was double digested with *Bam*HI plus *Eco*RI, cloned into the pGEM-T easy vector (Promega Corp., Madison, WI, USA), and named pGEM-Amh1-2. Using this plasmid as a template, four *amh* cDNA fragments were PCR amplified with the primer pairs amh10–amh14 (fragment a): Amh proprotein, the cleavage site, and an His_6_-tag); amh15–amh13 (fragment b): the cleavage site, an His_6_-tag and the mature Amh protein); amh10–amh17 (fragment c): Amh proprotein and the cleavage site; amh12–amh16 (fragment d): the cleavage site, the mature Amh protein, and an His_6_-tag preceded by a protease recognition sequence for His_6_-tag removal. Equal quantities of fragments (a) and (b) were joined in equal parts in an overlapping PCR reaction with primers amh10–amh13. Fragments (c) and (d) were fused in a PCR reaction using primers amh10–amh16. The resulting PCR products were double digested with *Avr*II and *Eco*RI, cloned into the pPIC9K *P. pastoris* expression plasmid (Invitrogen,) and termed pPICK9-His_6_Amh and pPICK9-AmhHis_6_, respectively. All the above PCR reactions were performed with the proofreading *Pfu*Ultra DNA polymerase (Agilent Technologies Inc., Santa Clara, CA, USA) following supplier guidelines and were further checked by sequencing. In general, the following conditions were used: initial denaturation at 94 °C for 1.5 min, followed by 30 cycles at 94 °C for 30 s, annealing temperature for 30 s, 72 °C for 1 min/kb, and a final extension of 10 min at 72 °C. When touchdown PCR [69] was used, the annealing was carried out at the highest temperature indicated for the first cycle, decreasing 0.5 °C each cycle until achieving the indicated minimum temperature, which was then maintained for the remaining cycles. Some of the used primers were designed to include 5′-overhangs with restriction enzyme sites for cloning purposes or no homologous overhangs. A touch-up PCR cycling protocol, consisting of an exact–opposite cycling mechanism of touch-down PCR, was adopted for PCRs using these primers.

The sequence modifications introduced in pPICK9-His_6_Amh were (Figure 1A) (1) deletion of the sequence coding for sea bass Amh amino acids position 1–22 corresponding to the putative signal peptide, so that the rest of the coding sequence could be cloned in the frame and downstream of the α-factor signal sequence in pPIC9K; (2) introduction of a Glu-Lys-Arg (EKR) site for cleavage of the Amh proprotein by the yeast prohormone-processing enzyme Kex2p by changing the Arg^426^-Ala-Thr-Arg-motif to a Glu^426^-Lys-Arg -motif replacing CGG GCC ACC AGA at nucleotide position 1313–1324 to GAG AAG CGA; (3) insertion of a His_6_-tag placed before Ala^430^ to facilitate purification of the mature peptide. Plasmid pPICK9-AmhHis_6_ had equivalent sequence modifications except (1) the His_6_-tag was placed at the end of the mature protein, before the stop codon; (2) an Ile-Glu-Gly-Arg cleavage site (IEGR site), placed N-terminal from the His_6_-tag, was added for the removal of the affinity tag by the Factor Xa protease.

### 4.3. Yeast Transformation and Screening for Mut^+^ and Muts Transformants

The *P. pastoris* yeast strain GS115 (his4) was purchased from Invitrogen. Prior to yeast transformation, pPICK9-His_6_Amh and pPICK9-AmhHis_6_ were linearized with the restriction endonuclease *Bgl*II to direct the integration of the expression cassette into the AOX1 gene locus, resulting in a methanol-utilization positive (Mu^+^) phenotype. The *P. pastoris* host strain was then transformed by electroporation according to the Invitrogen guidelines, using the ECM 830 Electroporation system (BTX; Holliston, MA, USA). Electroporated cells were spread on MD agar plates (1.34% yeast nitrogen base (YNB), 4 × 10^−5^% biotin, 2% dextrose, 1.5% agar) and incubated at 29 °C for 3 days. Colonies that grew on minimal methanol agar plates (1.34% YNB, 4 × 10^−5^% biotin, 0.5% methanol, 1.5% agar) plus YPD agar plates (1.0% yeast extract, 2% peptone, 2% dextrose, 1.5% agar) containing variable amounts of G418 sulfate (Gibco, Life Technologies™ Ltd., Paisley, Scotland, UK) (final concentrations of 0.5, 1.0 and 2.0 mg/mL) were selected as positive transformants. Colonies were further checked by PCR using DFS DNA Taq Polymerase (Bioron, GmbH, Römerberg, Germany) and primer pair 5′ AOX1 and 3′ AOX1 (Table S1), following the procedure indicated by the provider. Expression of the recombinant proteins was induced essentially as previously described [62]. In the initial tests, single selected colonies were grown in BMGY medium (1% yeast extract, 2% peptone, 1.34% YNB, 1% glycerol, 4 × 10^−5^% biotin, and 100 mM potassium phosphate, pH 6) with shaking (250 rpm) for 21 h at 29 °C. The cells were harvested by centrifugation at 2000× *g* for 5 min at room temperature (RT). To induce expression of the exogenous gene, cells were resuspended in a BMMY medium (BMGY with 0.5% methanol instead of 1% glycerol) using ¼ of the original culture volume. Incubation continued for another 72 h at 29 °C, adding methanol at a concentration of 0.5% every 24 h. Cultures were centrifuged at 15,000× *g* for 3 min at RT. The harvested supernatants and cell pellets were stored at −70 °C before being assayed by Western blot and their bioactivity was evaluated in a cell-based reporter assay that uses the specific sea bass Amhr2 [30]. GS115 cells transformed with empty pPICK9 vector and treated in the same manner were used as a control for endogenous expression.

### 4.4. Large Scale Production of Recombinant Sea Bass Amh in Yeast

Selected clones of *P. pastoris* expressing recombinant sea bass Amh or empty pPICK9 were grown in BMG medium (1.34% YNB, 1% glycerol, 4 × 10^−5^% biotin, and 100 mM potassium phosphate, pH 6) with 100 µg/mL of G418, and later induced in BMM medium (BMG with 0.5% methanol instead of 1% glycerol), all as described above. Harvested culture supernatants (~6 L) were ultrafiltered using Centricon Plus-70 centrifugal units (Millipore, Burlington, MA, USA), cut-off 3 kDa, following the manufacturer’s recommendations. Before loading the columns, media were centrifuged for 3 min at 2000× *g* to remove cell debris. The concentrated medium was frozen at −70 °C. Recombinant sea bass Amh_C_ was purified by immobilized metal affinity chromatography (IMAC Ni^2+^) from concentrated medium (60–70 mL) on 1 mL His GraviTrap Nickel Sepharose 6 Fast Flow prepacked columns (GE Healthcare Bio-Sciences, Chicago, IL, USA) in PBS (20 mM sodium phosphate, 500 mM NaCl, pH 7.4) containing 20 mM imidazole, according to the manufacturer’s recommendations. PBS containing 500 mM imidazole was used for the final elution step. Finally, the imidazole concentration was reduced to less than 100 mM with an Amicon PLBC Ultracel-3 kDa membrane filter unit (Millipore), in PBS (pH 7) and the protein fraction was kept frozen at −70 °C until further use.

### 4.5. Sea Bass Amhr2-Directed Antibodies

The rabbit antisera were produced on demand by Agrisera AB (Vännäs, Sweden). Anti-Amhr2 was raised against a synthetic peptide corresponding to amino acids I^67^NGQPQVDLLAC^78^ of sea bass Amhr2, located in the extracellular domain. The antibody was affinity-purified against the synthetic peptide used for the immunization protocol and its titer was tested by enzyme-linked immunosorbent assay.

### 4.6. Western Blot

For immunoblotting, different quantities of recombinant sea bass Amh, and protein extracts from CHO/HEK293/COS-7 cells transfected with Amhr2 or pcDNA3, previtellogenic ovaries and follicular cells were mixed with Laemmli sample buffer and distilled water, denatured at 95 °C for 5 min and subjected to 12–15% SDS-PAGE. After electrophoresis, the proteins were transferred onto previously activated PVDF membranes (Immobilon P, Millipore, Burlington, MA, USA) using the Trans-Blot Turbo™ Blotting System (Bio-Rad Laboratories, Inc., Hercules, CA, USA). The membranes were blocked for 1 h at room temperature in Tris-buffered saline with Tween 20 (TBST; 20 mM Tris, 140 mM NaCl, 0.1% Tween, pH 7.6) and blotting-grade blocker (5% non-fat milk powder; Bio-Rad). Then, membranes were incubated with the sea bass anti-C Amh antibody (1 μg/mL, [30]) or sea bass anti-Amhr2 antibody (2 µg/mL) overnight at 4 °C. Bound antibodies were detected with 1:25,000-diluted goat anti-rabbit IgG (Sigma-Aldrich, Inc., Saint Louis, MO, USA) coupled to horseradish peroxidase (HRP), and proteins were visualized by using enhanced chemiluminescence (Pierce™ ECL Plus Western Blotting Substrate) in the Amersham Imager 600 (GE Healthcare Bio-Sciences, Chicago, IL, USA). Quantification of band density was carried out using the ImageQuant™ TL software (GE Healthcare Bio-Sciences, Chicago, IL, USA). A known concentration of sea bass Amh_C_ produced in CHO cells [30] served as a standard curve for the semi quantification of *P. pastoris* sea bass Amh_C_ concentration.

### 4.7. Cell Culture, Transfection and Luciferase Assay

African green monkey kidney fibroblast-like (COS-7) cells were used to express the sea bass Amhr2 protein as previously described [30]. Cells were seeded onto 24 well plates (~1.5 × 10^5^ cells per well) in Dulbecco modified Eagle medium (DMEM) GlutaMAX (Life Technologies, Inc., Life Technologies™ Ltd., Paisley, Scotland, UK) supplemented with 10% *v*/*v* heat-inactivated FBS, and 100 U/mL of penicillin and streptomycin, at 37 °C in a 5% CO_2_ incubator. Cells were grown to 75–80% confluence and co-transfected using FuGENE^®^ HD Transfection Reagent (Promega) with the following plasmids: (i) the BRE-Luc reporter plasmid (100 ng), which has multiple optimized BMP-responsive elements driving the expression of the firefly luciferase gene [70], (ii) the pcDNA3-Amhr2 expression plasmid (415 ng) [30], and (iii) the pRL-TK reporter plasmid (Promega, Corp., Madison, WI, USA) (20 ng), which constitutively expresses the Renilla luciferase gene, to normalize transfection. Different amounts of the empty vector pBlueScript SK (Stratagene) were co-transfected to maintain the total amount of DNA constant in individual transfections. The following day, cells were plated onto 96-well plates. After 6–10 h, the medium was replaced with DMEM supplemented with 1% FBS containing different concentrations of purified sea bass AmhC (0.25, 0.5 and 1 µg/mL) or human AMH (hAMH) (0.05, 0.1 and 0.5 µg/mL; R&D Systems, Inc., Minneapolis, MN, USA). IMAC Ni^2+^—purified media obtained from *P. pastoris* expressing the empty pPIC9K vector served as a control for sea bass Amh, while PBS with 0.1% bovine serum albumin (BSA) and 4 mM HCl was used as a control for hAmh. After incubation at 37 °C for 24 h, the cells were washed twice with PBS pH 7.4 and harvested in 20 µL of Passive Reporter Lysis buffer (Promega). Luciferase activity was determined using the Dual-Luciferase Reporter Assay System (Promega), following the manufacturer’s instructions. The light emitted was measured in a luminometer (Berthold Junior; EG&G, Bad Wildbad, Germany) and expressed as relative light units (RLU). Firefly luciferase values were normalized to Renilla luciferase activity. The results are expressed relative to their control group (pPIC9K empty vector medium, or BSA-HCl medium).

### 4.8. Detection of Endogenous Amh and Amhr2 in Adult Sea Bass Ovaries by Immunohistochemistry

Ovarian samples from September (*n* = 1) (previtellogenesis) and November (*n* = 2) (vitellogenesis) were fixed with 4% paraformaldehyde (PFA) in PBS overnight at 4 °C and subsequently dehydrated and embedded in paraffin. Sections of approximately 5 µm thickness were deparaffinized in xylene, rehydrated in decreasing concentrations of ethanol, and washed twice with 0.1 M PBS. Antigen retrieval was carried out with Tris-EDTA Buffer (10 mM Tris Base, 1 mM EDTA, and 0.05% Tween 20, pH 9.0) at 95 °C for 15 min. After heat-induced antigen retrieval, slides were allowed to cool at RT, washed twice with PBST (0.1% Triton X-100), and blocked with 3% normal goat serum (NGS), 1% BSA in PBS for 1 h, before incubation overnight at 4 °C with 5 µg/mL of anti-C Amh primary antibody [30] or 5 µg/mL of anti-Amhr2 diluted in 3% NGS, 1% BSA in PBS. The sections were then washed twice in PBST and immersed in 0.5% hydrogen peroxide for 30 min to quench endogenous peroxidase activity. Slides were incubated for 1.5 h at RT with secondary goat anti-rabbit IgG- HRP conjugated (GAR-HRP, Bio-Rad) diluted 1:200 in 3% NGS, 1% BSA in PBS. As substrate for color development, 3,3′-Diaminobenzidine (DAB, ACROS Organics, Waltham, MA, USA) was used. Nuclei were counterstained with 12.5% hematoxylin (Sigma-Aldrich) for 30 s. Slides incubated without primary antibodies served as a negative control to check for any false positives arising from nonspecific interactions of the secondary antibody. Sections were examined and photographed with a Nikon Eclipse E600 imager microscope (Nikon Instruments Europe).

### 4.9. Ovary Tissue Culture

For in vitro tissue culture, previtellogenic ovaries of adult sea bass were used. Fish, sacrificed in October, were anesthetized with an overdose of ethyl 3-aminobenzoate methanesulfonate (MS-222; 300–400 mg/L; Sigma- Aldrich, St. Louis, MO, USA) and euthanized by decapitation in accordance with Spanish Royal Decree (53/2013) and European legislation (2010/63/EU) for the protection of animals used for experimentation. Ovaries were dissected and rapidly immersed in ice-cold Sea Bass Ringer (SBR) containing 0.5% BSA (fraction V, Sigma-Aldrich, Inc., Saint Louis, MO, USA), 100 units/mL penicillin/streptomycin and 250 ng/mL Amphotericin B (Life Technologies, Inc.). Ovarian tissue was dissected into small fragments on ice using a razor blade. Pieces of the tissue preparation were transferred to 96-well plates (about 15–20 mg per well) containing 0.1 mL of SBR. The explants were preincubated for 60 min at 21 °C in shaking conditions (100 rpm). After preincubation, the medium was replaced with 0.1 mL of fresh SBR containing different quantities of purified mature recombinant sea bass AmhC (0.25, 0.5 and 1 µg/mL), human AMH (Bio-Techne R&D Systems, Minneapolis, MN, USA; S.L.U.; 0.05, 0.1, 0.5 µg/mL) or control medium. After 24 h of incubation, under the same conditions as for preincubation, 300 ng/mL of seabass single-chain Fsh [63] or CHO control medium was added, and samples were further incubated for 24 h. After incubation, the medium was collected and gonadal explants were deep-frozen in liquid nitrogen and stored at −70 °C until RNA extraction. Four independent experiments using tissue from six different females were carried out.

### 4.10. Estradiol Immunoassay

The estradiol (E2) content of the culture medium was measured by a conventional enzyme immunoassay (EIA), validated for use on sea bass in our laboratory [63]. First, the culture medium was extracted with methanol, the organic solvent was evaporated and the dry extract was reconstituted in assay buffer (EIA buffer, Cayman Chemical;Ann Arbor, MI, USA) by vortexing. The assay was performed in a final volume of 150 μL in 96-well microtiter plates coated with mouse anti-rabbit IgG monoclonal antibodies (Clone RG-16, Sigma-Aldrich, Inc.). The components of the assay were (i) the E2 acetylcholinesterase conjugate (E-AChE, Cayman Chemical, Ann Arbor, MI, USA) used as tracer (0.083 UE/mL), (ii) a specific anti-E2 rabbit antiserum [71] (diluted to 1:2,500,000), (iii) E2 standards (ranging from 80 ng/mL to 0.039 ng/mL), or samples, were added in a volume of 50 μL. Plates were incubated overnight at 37 °C, rinsed, and color development was performed by the addition of 200 µL/well of Ellman’s reagent followed by incubation under gentle agitation for 2 h at 20 °C in the dark. Optical density was read at 405 nm in a microplate reader (Bio-Rad microplate reader model 3550). The sensitivity of the assay was 0.156 ng/mL (Bi/B0 = 90%) and half-displacement (Bi/B0 = 50%) occurred around 1.90 ng/mL.

### 4.11. Quantitative Real-Time PCR (qPCR)

The expression of *cyp19a1a* in treated and non-treated ovarian explants and the expression of *amh* and *amhr2* in annual samples of follicular cells were determined by qPCR. Total RNA was extracted from ±15 mg of ovary tissue and follicular cells using the Maxwell™ 16 LEV simplyRNA Tissue Kit (Promega Corp., Madison, WI, USA) on a Maxwell™ 16 Instrument (Promega Corp.). All the RNA samples were checked to be free of genomic DNA. For cDNA synthesis, 1 μg of total RNA was reverse-transcribed using Superscript III (Invitrogen Corp., Carlsbad, CA, USA) and random hexamers as primers following the manufacturer’s instructions. As an internal control, 0.4 ng of mRNA from the *luciferase* (*luc1*) gene (luL4561, Promega) was added to each reverse transcription reaction. All qPCR assays were run in duplicate for each sample on 96-well plates using the CFX384 Touch™ Real-Time PCR Detection System (Bio-Rad Laboratories, Inc., Hercules, CA, USA) with default settings for the fluorescence used detection system. Data were captured and analyzed with CFX Manager™ Software (version 3.1). For each reaction, optimized amounts of primers and probes [21,30,61] (Table S2) were mixed with 1 μL of non-diluted cDNA and 1× PyroTaq PROBE qPCR Mix Plus (Cultek) in a final reaction volume of 20 μL. Expression of the ribosomal protein L13a (*rpl13a*) [72] gene or *luciferase* gene (Table S2) in 1:50 diluted cDNA samples were used as reference genes for normalization of data from cDNA. The expression of *rpl13a* was stable among the different samples and treatments. Tenfold serial dilutions of known concentrations of plasmids containing the genes of interest were included as a standard curve. The average value for correlation coefficients (R2) of the standard curves was 0.99. PCR efficiencies ranged from 91% to 98%.

### 4.12. Statistical Analyses

Data are shown as the mean ± SEM and were statistically analyzed using one-way ANOVA followed by the Tukey multiple comparison method using GraphPad Prism (GraphPad Software, Inc., La Jolla, CA, USA). When the test of equal variance failed, ANOVA on ranks (Kruskal-Wallis non-parametric test) was performed followed by a pairwise multiple comparison procedure (i.e., the Dunn method). Criteria for significance were set at a *p*-value of <0.05.

## 5. Conclusions

We suggest a role for Amh in early vitellogenesis, during which it locally regulates ovarian steroidogenesis and produces an additive increase in the subsequent endocrine effect of Fsh during vitellogenesis. However, these results need to be studied in-depth and could differ from those obtained in studies of earlier ovarian stages or in other teleost species, as already observed with the different expression patterns of *amh* and aromatase during oogenesis.

## Supplementary Materials

The following are available online at https://www.mdpi.com/article/10.3390/ijms221810092/s1.

## References

[1] Adolfi M.C., Nakajima R.T., Nóbrega R.H., Schartl M. (2019). Intersex, Hermaphroditism, and Gonadal Plasticity in Vertebrates: Evolution of the Müllerian Duct and Amh/Amhr2 Signaling. Annu. Rev. Anim. Biosci..

[2] Di Clemente N., Jamin S.P., Lugovskoy A., Carmillo P., Ehrenfels C., Picard J.-Y., Whitty A., Josso N., Pepinsky R.B., Cate R.L. (2010). Processing of Anti-Müllerian Hormone Regulates Receptor Activation by a Mechanism Distinct from TGF-β. Mol. Endocrinol..

[3] Teixeira J., He W.W., Shah P.C., Morikawa N., Lee M.M., Catlin E.A., Hudson P.L., Wing J., Maclaughlin D.T., Donahoe P.K. (1996). Developmental expression of a candidate müllerian inhibiting substance type II receptor. Endocrinology.

[4] Wang P.-Y., Protheroe A., Clarkson A.N., Imhoff F., Koishi K., McLennan I.S. (2009). Müllerian inhibiting substance contributes to sex-linked biases in the brain and behavior. Proc. Natl. Acad. Sci. USA.

[5] Ricci M., Mohapatra B., Urbiztondo A., Birusingh R.J., Morgado M., Rodriguez M.M., Lincoln J., Vatta M. (2010). Differential Changes in TGF-β/BMP Signaling Pathway in the Right Ventricular Myocardium of Newborns with Hypoplastic Left Heart Syndrome. J. Card. Fail..

[6] Catlin E.A., Powell S.M., Manganaro T.F., Hudson P.L., Ragin R.C., Epstein J., Donahoe P.K. (1990). Sex-specific Fetal Lung Development and Müllerian Inhibiting Substance. Am. Rev. Respir. Dis..

[7] Jamin S.P., Arango N.A., Mishina Y., Hanks M.C., Behringer R.R. (2002). Requirement of Bmpr1a for Müllerian duct regression during male sexual development. Nat. Genet..

[8] Di Clemente N., Josso N., Gouédard L., Belville C. (2003). Components of the anti-Müllerian hormone signaling pathway in gonads. Mol. Cell. Endocrinol..

[9] McLennan I.S., Pankhurst M.W. (2015). Anti-Müllerian hormone is a gonadal cytokine with two circulating forms and cryptic actions. J. Endocrinol..

[10] Baarends W.M., Uilenbroek J.T., Kramer P., Hoogerbrugge J.W., van Leeuwen E.C., Themmen A.P., Grootegoed J.A. (1995). Anti-müllerian hormone and anti-müllerian hormone type II receptor messenger ribonucleic acid expression in rat ovaries during postnatal development, the estrous cycle, and gonadotropin-induced follicle growth. Endocrinology.

[11] Baarends W.M., Hoogerbrugge J.W., Post M., Visser J.A., De Rooij D.G., Parvinen M., Themmen A.P., Grootegoed J.A. (1995). Anti-müllerian hormone and anti-müllerian hormone type II receptor messenger ribonucleic acid expression during postnatal testis development and in the adult testis of the rat. Endocrinology.

[12] Poole D.H., Ocón-Grove O.M., Johnson A.L. (2016). Anti-Müllerian hormone (AMH) receptor type II expression and AMH activity in bovine granulosa cells. Theriogenology.

[13] Josso N., Picard J.Y., Rey R., di Clemente N. (2006). Testicular anti-Müllerian hormone: History, genetics, regulation and clinical applications. Pediatr. Endocrinol. Rev. PER.

[14] Visser J.A., Themmen A.P.N. (2005). Anti-Müllerian hormone and folliculogenesis. Mol. Cell. Endocrinol..

[15] Vigier B., Forest M.G., Eychenne B., Bezard J., Garrigou O., Robel P., Josso N. (1989). Anti-Mullerian hormone produces endocrine sex reversal of fetal ovaries. Proc. Natl. Acad. Sci. USA.

[16] Rouiller-Fabre V., Carmona S., Merhi R.A., Cate R., Habert R., Vigier B. (1998). Effect of anti-Mullerian hormone on Sertoli and Leydig cell functions in fetal and immature rats. Endocrinology.

[17] Rey R., Lukas-Croisier C., Lasala C., Bedecarrás P. (2003). AMH/MIS: What we know already about the gene, the protein and its regulation. Mol. Cell. Endocrinol..

[18] Durlinger A.L.L., Gruijters M.J.G., Kramer P., Karels B., Ingraham H.A., Nachtigal M.W., Uilenbroek J.T.J., Grootegoed J.A., Themmen A.P.N. (2002). Anti-Müllerian hormone inhibits initiation of primordial follicle growth in the mouse ovary. Endocrinology.

[19] Miura T., Miura C., Konda Y., Yamauchi K. (2002). Spermatogenesis-preventing substance in Japanese eel. Development.

[20] Rodríguez-Marí A., Yan Y.-L., Bremiller R.A., Wilson C., Cañestro C., Postlethwait J.H. (2005). Characterization and expression pattern of zebrafish Anti-Müllerian hormone (Amh) relative to sox9a, sox9b, and cyp19a1a, during gonad development. Gene Expr. Patterns GEP.

[21] Halm S., Rocha A., Miura T., Prat F., Zanuy S. (2007). Anti-Müllerian hormone (AMH/AMH) in the European sea bass: Its gene structure, regulatory elements, and the expression of alternatively-spliced isoforms. Gene.

[22] Klüver N., Pfennig F., Pala I., Storch K., Schlieder M., Froschauer A., Gutzeit H.O., Schartl M. (2007). Differential expression of anti-Müllerian hormone (amh) and anti-Müllerian hormone receptor type II (amhrII) in the teleost medaka. Dev. Dyn. Off. Publ. Am. Assoc. Anat..

[23] Pfennig F., Standke A., Gutzeit H.O. (2015). The role of Amh signaling in teleost fish--Multiple functions not restricted to the gonads. Gen. Comp. Endocrinol..

[24] Yoshinaga N., Shiraishi E., Yamamoto T., Iguchi T., Abe S., Kitano T. (2004). Sexually dimorphic expression of a teleost homologue of Müllerian inhibiting substance during gonadal sex differentiation in Japanese flounder, *Paralichthys olivaceus*. Biochem. Biophys. Res. Commun..

[25] Wang X.G., Orban L. (2007). Anti-Müllerian hormone and 11 beta-hydroxylase show reciprocal expression to that of aromatase in the transforming gonad of zebrafish males. Dev. Dyn. Off. Publ. Am. Assoc. Anat..

[26] Maugars G., Schmitz M. (2008). Gene expression profiling during spermatogenesis in early maturing male Atlantic salmon parr testes. Gen. Comp. Endocrinol..

[27] Morinaga C., Saito D., Nakamura S., Sasaki T., Asakawa S., Shimizu N., Mitani H., Furutani-Seiki M., Tanaka M., Kondoh H. (2007). The hotei mutation of medaka in the anti-Mullerian hormone receptor causes the dysregulation of germ cell and sexual development. Proc. Natl. Acad. Sci. USA.

[28] Nakamura S., Watakabe I., Nishimura T., Picard J.-Y., Toyoda A., Taniguchi Y., di Clemente N., Tanaka M. (2012). Hyperproliferation of mitotically active germ cells due to defective anti-Müllerian hormone signaling mediates sex reversal in medaka. Development.

[29] Pala I., Klüver N., Thorsteinsdóttir S., Schartl M., Coelho M.M. (2008). Expression pattern of anti-Müllerian hormone (amh) in the hybrid fish complex of *Squalius alburnoides*. Gene.

[30] Rocha A., Zanuy S., Gómez A. (2016). Conserved anti-müllerian hormone: Anti-müllerian hormone type-2 receptor specific interaction and intracellular signaling in teleosts. Biol. Reprod..

[31] Zhang G., Wang W., Su M., Zhang J. (2018). Effects of recombinant gonadotropin hormones on the gonadal maturation in the spotted scat, *Scatophagus argus*. Aquaculture.

[32] Skaar K.S., Nóbrega R.H., Magaraki A., Olsen L.C., Schulz R.W., Male R. (2011). Proteolytically activated, recombinant anti-mullerian hormone inhibits androgen secretion, proliferation, and differentiation of spermatogonia in adult zebrafish testis organ cultures. Endocrinology.

[33] Lin Q., Mei J., Li Z., Zhang X., Zhou L., Gui J.-F. (2017). Distinct and Cooperative Roles of amh and dmrt1 in Self-Renewal and Differentiation of Male Germ Cells in Zebrafish. Genetics.

[34] Yan Y.-L., Batzel P., Titus T., Sydes J., Desvignes T., BreMiller R., Draper B., Postlethwait J.H. (2019). A Hormone That Lost Its Receptor: Anti-Müllerian Hormone (AMH) in Zebrafish Gonad Development and Sex Determination. Genetics.

[35] Liu X., Xiao H., Jie M., Dai S., Wu X., Li M., Wang D. (2020). Amh regulate female folliculogenesis and fertility in a dose-dependent manner through Amhr2 in Nile tilapia. Mol. Cell. Endocrinol..

[36] Zhang Z., Zhu B., Chen W., Ge W. (2020). Anti-Müllerian hormone (Amh/amh) plays dual roles in maintaining gonadal homeostasis and gametogenesis in zebrafish. Mol. Cell. Endocrinol..

[37] Fairlie W.D., Zhang H., Brown P.K., Russell P.K., Bauskin A.R., Breit S.N. (2000). Expression of a TGF-beta superfamily protein, macrophage inhibitory cytokine-1, in the yeast *Pichia pastoris*. Gene.

[38] Papakonstantinou T., Harris S.J., Fredericks D., Harrison C., Wallace E.M., Hearn M.T.W. (2009). Synthesis, purification and bioactivity of recombinant human activin A expressed in the yeast *Pichia pastoris*. Protein Expr. Purif..

[39] Fredericks D., Clay R., Warner T., O’Connor A., de Kretser D.M., Hearn M.T.W. (2010). Optimization of the expression of recombinant human activin A in the yeast *Pichia pastoris*. Biotechnol. Prog..

[40] Pulkki M.M., Myllymaa S., Pasternack A., Lun S., Ludlow H., Al-Qahtani A., Korchynskyi O., Groome N., Juengel J.L., Kalkkinen N. (2011). The bioactivity of human bone morphogenetic protein-15 is sensitive to C-terminal modification: Characterization of the purified untagged processed mature region. Mol. Cell. Endocrinol..

[41] Pépin D., Hoang M., Nicolaou F., Hendren K., Benedict L.A., Al-Moujahed A., Sosulski A., Marmalidou A., Vavvas D., Donahoe P.K. (2013). An albumin leader sequence coupled with a cleavage site modification enhances the yield of recombinant C-terminal Mullerian Inhibiting Substance. Technology.

[42] Wu G.-C., Li H.-W., Luo J.-W., Chen C., Chang C.-F. (2015). The Potential Role of Amh to Prevent Ectopic Female Development in Testicular Tissue of the Protandrous Black Porgy, *Acanthopagrus schlegelii*. Biol. Reprod..

[43] Macauley-Patrick S., Fazenda M.L., McNeil B., Harvey L.M. (2005). Heterologous protein production using the *Pichia pastoris* expression system. Yeast.

[44] Goh J.B., Ng S.K. (2018). Impact of host cell line choice on glycan profile. Crit. Rev. Biotechnol..

[45] Bézard J., Vigier B., Tran D., Mauléon P., Josso N. (1987). Immunocytochemical study of anti-Müllerian hormone in sheep ovarian follicles during fetal and post-natal development. J. Reprod. Fertil..

[46] Weenen C., Laven J.S.E., Von Bergh A.R.M., Cranfield M., Groome N.P., Visser J.A., Kramer P., Fauser B.C.J.M., Themmen A.P.N. (2004). Anti-Müllerian hormone expression pattern in the human ovary: Potential implications for initial and cyclic follicle recruitment. Mol. Hum. Reprod..

[47] Stubbs S.A., Hardy K., Da Silva-Buttkus P., Stark J., Webber L.J., Flanagan A.M., Themmen A.P.N., Visser J.A., Groome N.P., Franks S. (2005). Anti-müllerian hormone protein expression is reduced during the initial stages of follicle development in human polycystic ovaries. J. Clin. Endocrinol. Metab..

[48] Rocha R.M.P., Lima L.F., Carvalho A.A., Chaves R.N., Bernuci M.P., Rosa-e-Silva A.C.J.S., Rodrigues A.P.R., Campello C.C., Figueiredo J.R. (2016). Immunolocalization of the Anti-Müllerian Hormone (AMH) in Caprine Follicles and the Effects of AMH on In Vitro Culture of Caprine Pre-antral Follicles Enclosed in Ovarian Tissue. Reprod. Domest. Anim..

[49] Modi D., Bhartiya D., Puri C. (2006). Developmental expression and cellular distribution of Mullerian inhibiting substance in the primate ovary. Reproduction.

[50] Schmidt K.L.T., Kryger-Baggesen N., Byskov A.G., Andersen C.Y. (2005). Anti-Müllerian hormone initiates growth of human primordial follicles in vitro. Mol. Cell. Endocrinol..

[51] Han Y., Peng C., Wang L., Guo J., Lu M., Chen J., Liu Y., Li S., Zhao M., Zhang Y. (2018). Female-to-male sex reversal in orange-spotted grouper (*Epinephelus coioides*) caused by overexpressing of Amh in vivo. Biol. Reprod..

[52] Von Schalburg K.R., Gowen B.E., Rondeau E.B., Johnson N.W., Minkley D.R., Leong J.S., Davidson W.S., Koop B.F. (2013). Sex-specific expression, synthesis and localization of aromatase regulators in one-year-old Atlantic salmon ovaries and testes. Comp. Biochem. Physiol. Part B Biochem. Mol. Biol..

[53] Ingraham H.A., Hirokawa Y., Roberts L.M., Mellon S.H., McGee E., Nachtigal M.W., Visser J.A. (2000). Autocrine and paracrine Müllerian inhibiting substance hormone signaling in reproduction. Recent Prog. Horm. Res..

[54] McGee E.A., Smith R., Spears N., Nachtigal M.W., Ingraham H., Hsueh A.J. (2001). Müllerian inhibitory substance induces growth of rat preantral ovarian follicles. Biol. Reprod..

[55] Cheon K.Y., Chung Y.J., Cho H.H., Kim M.R., Cha J.H., Kang C.S., Lee J.Y., Kim J.H. (2018). Expression of Müllerian-Inhibiting Substance/Anti-Müllerian Hormone Type II Receptor in the Human Theca Cells. J. Clin. Endocrinol. Metab..

[56] Von Hofsten J., Larsson A., Olsson P.E. (2005). Novel steroidogenic factor-1 homolog (ff1d) is coexpressed with anti-Mullerian hormone (AMH) in zebrafish. Dev. Dyn. Off. Publ. Am. Assoc. Anat..

[57] Morais R.D.V.S., Crespo D., Nóbrega R.H., Lemos M.S., van de Kant H.J.G., de França L.R., Male R., Bogerd J., Schulz R.W. (2017). Antagonistic regulation of spermatogonial differentiation in zebrafish (*Danio rerio*) by Igf3 and Amh. Mol. Cell. Endocrinol..

[58] Fernandino J.I., Hattori R.S., Kimura H., Strüssmann C.A., Somoza G.M. (2008). Expression profile and estrogenic regulation of anti-Müllerian hormone during gonadal development in pejerrey *Odontesthes bonariensis*, a teleost fish with strong temperature-dependent sex determination. Dev. Dyn. Off. Publ. Am. Assoc. Anat..

[59] Johnsen H., Tveiten H., Torgersen J.S., Andersen Ø. (2013). Divergent and sex-dimorphic expression of the paralogs of the Sox9-Amh-Cyp19a1 regulatory cascade in developing and adult atlantic cod (*Gadus morhua* L.). Mol. Reprod. Dev..

[60] Luckenbach J.A., Iliev D.B., Goetz F.W., Swanson P. (2008). Identification of differentially expressed ovarian genes during primary and early secondary oocyte growth in coho salmon, *Oncorhynchus kisutch*. Reprod. Biol. Endocrinol. RBE.

[61] Rocha A., Zanuy S., Carrillo M., Gómez A. (2009). Seasonal changes in gonadal expression of gonadotropin receptors, steroidogenic acute regulatory protein and steroidogenic enzymes in the European sea bass. Gen. Comp. Endocrinol..

[62] Molés G., Gómez A., Carrillo M., Zanuy S. (2012). Development of a homologous enzyme-linked immunosorbent assay for European sea bass FSH. Reproductive cycle plasma levels in both sexes and in yearling precocious and non-precocious males. Gen. Comp. Endocrinol..

[63] Molés G., Zanuy S., Muñoz I., Crespo B., Martínez I., Mañanós E., Gómez A. (2011). Receptor Specificity and Functional Comparison of Recombinant Sea Bass (*Dicentrarchus labrax*) Gonadotropins (Fsh and Lh) Produced in Different Host Systems1. Biol. Reprod..

[64] Durlinger A.L., Gruijters M.J., Kramer P., Karels B., Kumar T.R., Matzuk M.M., Rose U.M., de Jong F.H., Uilenbroek J.T., Grootegoed J.A. (2001). Anti-Müllerian hormone attenuates the effects of FSH on follicle development in the mouse ovary. Endocrinology.

[65] Durlinger A.L., Kramer P., Karels B., de Jong F.H., Uilenbroek J.T., Grootegoed J.A., Themmen A.P. (1999). Control of primordial follicle recruitment by anti-Müllerian hormone in the mouse ovary. Endocrinology.

[66] Grossman M.P., Nakajima S.T., Fallat M.E., Siow Y. (2008). Müllerian-inhibiting substance inhibits cytochrome P450 aromatase activity in human granulosa lutein cell culture. Fertil. Steril..

[67] Crespo B., Zanuy S., Gómez A. (2013). Development of an in vitro system for functional studies of ovarian follicular cells in European sea bass (*Dicentrarchus labrax*). Cytotechnology.

[68] Asturiano J.F., Sorbera L.A., Ramos J., Kime D.E., Carrilo M., Zanuy S. (2000). Hormonal regulation of the European sea bass reproductive cycle: An individualized female approach. J. Fish Biol..

[69] Don R.H., Cox P.T., Wainwright B.J., Baker K., Mattick J.S. (1991). “Touchdown” PCR to circumvent spurious priming during gene amplification. Nucleic Acids Res..

[70] Korchynskyi O., ten Dijke P. (2002). Identification and functional characterization of distinct critically important bone morphogenetic protein-specific response elements in the Id1 promoter. J. Biol. Chem..

[71] Prat F., Zanuy S., Carrillo M., de Mones A., Fostier A. (1990). Seasonal changes in plasma levels of gonadal steroids of sea bass, *Dicentrarchus labrax* L.. Gen. Comp. Endocrinol..

[72] Mitter K., Kotoulas G., Magoulas A., Mulero V., Sepulcre P., Figueras A., Novoa B., Sarropoulou E. (2009). Evaluation of candidate reference genes for QPCR during ontogenesis and of immune-relevant tissues of European seabass (*Dicentrarchus labrax*). Comp. Biochem. Physiol. Part B Biochem. Mol. Biol..

