# Supplementary material for: Functional Activity of Recombinant Forms of Amh and Synergistic Action with Fsh in European Sea Bass Ovary
Source: Int J Mol Sci. 2021 Sep 18;22(18):10092. doi: 10.3390/ijms221810092 (PMC8467395; doi:10.3390/ijms221810092)
Supplement: Supplementary file 1 [file ijms-22-10092-s001.zip › ijms-1346840-supplementary.pdf]

Supplementary Materials

```
                                     *       420         *       440         *       460         *       480
Homo sapiens               : SAELEESPPSADPFLETLTRLVRALRVP------------PARASAPRLALDPDALAGFPQGLVNLS-DPAALERLLDGE : 343
Mus musculus               : SLEPEALPHSADPFLETLTRLVRALRGP------------LTQASNTQLALDPGALASFPQGLVNLS-DPAALGRLLDWE : 340
Gallus gallus              : ANTTSPTPGDSGQFLGVLTLFIRRVLSPRS---------EPPTQPSSQHWLDFQMMETLPHQLLNLS-ETAALERLVQSE : 430
Alligator sinensis         : ASTSTLAPGSTDQFLGSLTRFVSGILGSSG---------EPLPASRVHHWLDVETAETLPHQQLNLS-EEAALERLVQSE : 456
Xenopus laevis             : HINSATVAEKKDGFLEMISQFSVLLLNSNG---------KAASTIKLPLDPNDHSVGDLYPQLFNVT-DVEALEWLVDSN : 377
Protopterus annectens      : HAASAVHRSTHELFLNHLRSFLSLVLEDSH---------DPPS--NSVLHLQQEALTALPHQLLNIS-DPTIIQMLVSSE : 460
Latimeria menadoensis      : ----------------------------------------S--KTVLE------EKLSHWDL---------------- :  14
Callorhinchus milii        : HHPGPSGRVSPRDFLRHLNSFLNMVLDYST---------DWRS--KSIFHLDKETIESLPHQSLNIS-NIYALELLVELE : 437
Acipenser baerii           : SIPPPHHWDSTYEFLKELKGFLSAMLRGENEGPPLQLHVDLDSLQSLPHGYLDSLQSLPHGYLNASSEDVALLRQLLESH : 396
Gasterosteus aculeatus     : NSS---PASSQTSFLCELKRFLGDVLP--------------QDLRESSPLQLDSLKSLPPLTLGPSS-SDTLLAGLINSS : 315
Dicentrarchus labrax       : VSGWSLASSQTSSFLCELKRFLGDVLP--------------QAHPESPKLQLTTLQSLPPLTLGSSS-SETLLAGLMNSS : 323
Oreochromis niloticus      : GSP---PASLQTSFLCEMKRFLGAVLP--------------QEHFTSPPLPLDSLQSLPPLSLGLSS-SETLLAVMINST : 304
Acanthopagrus schlegelii   : GS----GSSQTSSFLCELKRFLGDVMP--------------QGQPESPPLQLTSLQSMPPLTLGVSS-SETLLAGLINSS : 318
Epinephelus coioides       : AS-------SHTSFFCELKRFLGDVLP--------------QDHSASPPLQLDSLQSLPPLMLGLSS-SETLLAGLINSS : 308
Monopterus albus           : VSSPSPASSQTLSFLCELKRFLGDILP--------------QEHPKPPLVHLDSLQSLPPLALGLSS-SETLLAELISSS : 338
Oncorhynchus mykiss        : KLSPSPAPSGTYTFLCELQKFLSDVMPPQ-----------TQSQPWAASVPLYSLDSLPPLSLGVSS-SETLLARLLNSS : 358
Oryzias latipes            : FGS--SGVPVQTSFVCELRRFLEGVLP--------------QDQDKQPLLQLDSLQALPPLPLGLSS-SETLLAALINSS : 289
Takifugu rubripes          : QTS---------SFLCELKQFLAGVTP--------------HSHPESPLLQLSSLQSLPPLTLGRST-SEATLAALIHSP : 283
Danio rerio                : IPQDERLPSRTYLFLCELQKFLRDILPQSK---------STTPQDDPSAVSLDTLHSLPPLRLGVSS-TESLLSGLVNSS : 336
```

Amh$_N$

```
                                     *       500         *       520         *       540         *       560
Homo sapiens               : EPLLLLLRPTAATTGD-PAPLHDPTSAPWATALARRVAAELQAAAAELRSLPGLPPATAP-LLARLLALC---------- : 411
Mus musculus               : EPLLLLLSPTAATERE-PIRLHGPASAPWAAGLQRRVAVELQAAASELRDLPGLPPTAPP-LLARLLALC---------- : 408
Gallus gallus              : EPSVLLFPQEGGAGLE-QHFGDWQPEG----TVLQLLMGKLQAVIHELRDIPEFQANTG--LFQHLLSFCYH-------- : 495
Alligator sinensis         : EPLLLLFPHNSQALLE-QHFRHWRVEG----RVLQLLLEELQAVIQDLRELPAFQANAG--LFQHLLAFCYY-------- : 521
Xenopus laevis             : DPLVFLFLSGNK-----FLQGTKVLQEKLAGKLLDKMTDKLQEVLEDMEEL--LSNKDHAQILRHLLHSCHGSYNVSYLP : 450
Protopterus annectens      : SPLIFMFPRKSK------SEWGQFLQWDLKGELLAMLTNRLQIVIEEVKEFPVFQNNETWLKLSQLLNYCYQSTNR---- : 530
Latimeria menadoensis      : -----------------------------QGKVVELLSARLQSVLQQVQEISSFQNRKVLLKLRHLLNHCYHSFNLTSLL :  65
Callorhinchus milii        : EPLVFLLPENAEVFLEDTSGENSGESRKQEEKIQRLFLTKMQGVIAQVLAIPVFQKEKVLKKLRYLLNQCNYPFPLPQVP : 517
Acipenser baerii           : TPTLFRFPRRLEDLRG--HRVELVLEADLLDLLNTRLTDTVAQIQRQMELQNHGGLPQLQRLLRGCFHLTQGRGVP---- : 470
Gasterosteus aculeatus     : SITVFSFRSRASGFQV--HSGELAFSPELLEELRRRLEAEVVKITAVMREEQVGRGATERLGRLRELSALPMM------- : 386
Dicentrarchus labrax       : ALTIFDFTNWGSMFQV--HHGELALSPALLEELRQRLEQTVLHTMEVIREEDVGHRATERLGRLKQLSAFPEK------- : 394
Oreochromis niloticus      : APTVFGFTSWGSVLPV--CHGELALSAALLEELRQRLDQTLVQMTEIIREEEVSLGAKESLGRLKELSALQEK------- : 375
Acanthopagrus schlegelii   : ALTIFSFSTCCSVSQV--HRRELALSPALAEELGQRLEQTMMQITEVIREEDVGHRATERLGRLRELSAFPKE------- : 389
Epinephelus coioides       : SPTIFSFTGCFSMFQT--HDGELALSPALLEEVKRRLEQTVVQLMEVIMEEEVGHRAAERLGRLRDLSAFPKT------- : 379
Monopterus albus           : APTVFSFTSWHSMFHV--YHGELALSPALLAELRQRLQQTVTQIREVIREEEVDRSATERLGRLEEVSVLLKK------- : 409
Oncorhynchus mykiss        : APTLFSFPTQGSVLRG--HRGELSLQPALLEVLRQRLEEVLVQM----RVEEVGKAGMDRLRRLQELSVLPKEGEE---- : 428
Oryzias latipes            : APTIFYFNNWGSKSQG--LRGQLILSPALLVELRLRLEQSEMLILDLAEQETVSPRTTARLERLKDLSASQQM------- : 360
Takifugu rubripes          : SLTVLSFGGCCSSSQV--HRGELALPPPLSEELRQKLEQTVTHITEAIR-EEVGDEAIKRLGRLVELSAFPRK------- : 353
Danio rerio                : TPTVFVFPQRQQGLQT--HRVEVTLDSPLLSVLRMRLDEAMAQVK----QQEAGRKMIDRLQKLTELSALSPDGED---- : 406
```

Amh$_N$

```
                                     *       580         *       600         *       620         *       640
Homo sapiens               : PGGPGGLG-------------DPLRALLLLKALQGLRVEWRGRDPRGPGRAQRSAGATAADGPCALRELSVDLR---AER : 475
Mus musculus               : PNDSRSSG-------------DPLRALLLLKALQGLRAEWHGREGRGRTRAQR--GDKGQDGPCALRELSVDLR---AER : 470
Gallus gallus              : PPGPAAGG--------QPPGSGKLHALLLLKALQTVRARWQER--RKVLRQNR---SARHQAHCRLQELTIDLR---DRN : 559
Alligator sinensis         : PPGPGSSDSLVTPLPEELRGPRQLHTLLLLKALQTVRTHWRER--RKVLWQNR---SARHQASCRLRELTVDIS---HYK : 593
Xenopus laevis             : SEAEN-------ISQVGESQNRKLHSLMLLKVLQTIRSYWQDQ--KKLSRRNR-GTG--TKPYCRLQELTISLKPFAEYK : 518
Protopterus annectens      : HSPTVEHDSELPSSITK-RRERKLHSFLLLKVLHTAKAFWIKK--LKLSRENR-NVN---NEYCRLHQLYISLK---YEG : 600
Latimeria menadoensis      : NIPASQSDD--AAKLNQNSKQRQIHTLLLLKVLQTIRAFWHKK--QKLSRESR-SATSNDESYCRLRELNVDLS---HET : 137
Callorhinchus milii        : QVPTRVGDAPKPGQRERNRKQKTYHTFLLLKTLQTVKTFWDKK--QKLFRQNR-SAG--NRSVCKLEELSIDFERL-SYD : 591
Acipenser baerii           : -----------PEEMPANPRQRQYHSLLLLKALQTVLSAWDQRGQRQKPTRQD--RSQEHHTDCGLRELSVSLLEY---K : 534
Gasterosteus aculeatus     : ---------------EPAPGGRQYRAFLLLKALQTAARAYEAQRGQRATRAGP--DITARANKCGLKSLSVTLD-----R : 444
Dicentrarchus labrax       : ---------------EPAAGESQYCAFLLLKALKTVARTYEVQRGLRATRADP--NNPVRGHTCGLRSLTVSLE-----K : 452
Oreochromis niloticus      : ---------------EHATGGSQFRVFLLLKALQTVAQTYDAQRKLRATRADP--SSSVRGGVCGLKALTVSLT-----K : 433
Acanthopagrus schlegelii   : ---------------EPATGESQYRAFLLLRALQTVARTYVLKRGQRTARAGP--SNPGGANICGLRSLTVSLE-----R : 447
Epinephelus coioides       : ---------------EPAAGGSQYRAFLLLKALQTVAGAYEVQRGLRATRAGP--NNPARGNLCGLRSLTVSLE-----K : 437
Monopterus albus           : ---------------EPAAGETQYRALLLLKALQTVTCAYELRRGLRATRADP--SNPVRDDGCGLKSLTVSLV-----K : 467
Oncorhynchus mykiss        : -----------APAGVGSPSETQYRALLLLKALQTVVGAWDVERGQRAARAGQ--KGPGNRHLCRLHSLTVSLE-----K : 490
Oryzias latipes            : ---------------EAA--ESHFCAFLLLKALKTVAHAYDMTRRLRSTRAGP--DSPPKGNICGLRSLTVSFE-----K : 416
Takifugu rubripes          : ---------------DLATGQSQYRAFLLLKALQTVSRTYTVKRGLRAIRANA--DTPARQKHCRLRSFTMSLE-----R : 411
Danio rerio                : -----------SEAATKDHKEAQYRSVLLLKALQMVLSNWESERAQRAARADE--DGPSASNQCHLQSLSVSLR-----K : 468
```

Amh$_N$ **Amh$_C$**

Figure S1. Amino acid sequence alignment showing part of the AmhN domain and the canonical cleavage site of Amh. GenBank/ UniProtKB/ Ensembl accession numbers: *Homo sapiens*, AAC25614.1; *Mus musculus*, CAA44912.1; *Gallus gallus*, NP_990361.1; *Alligator sinensis*, XP_006037226.1; *Xenopus laevis*, BAO04196.1; *Protopterus annectens*, AWT24621.1; *Latimeria menadoensis* (partial),

CCP19124.1; *Callorhinchus milii*, XP_007896900.1; *Acipenser baerii*, AKE47491.1; *Gasterosteus aculeatus*, G3PGH3; *Dicentrarchus labrax*, CAJ78431.1; *Oreochromis niloticus*, XP_013130583.1; *Acanthopagrus schlegelii*, ADB22521.1; *Epinephelus coioides*, AJW76790.1; *Monopterus albus*, AHF27393.1; *Oncorhynchus mykiss*, CDQ57255.1; *Oryzias latipes*, ABG24272.1; *Takifugu rubripes*, H2V975**;** *Danio rerio*, NP_001007780.1. The numbers on the right refer to amino acid position. Light blue font color indicates a proprotein convertase subtilisin/kexin type 2 cleavage site predicted by iProt-Sub server that could not be proven. Mandarine font color exposes a conserved N-glycosylation site. The primary proteolytic cleveage sites in human AMH (Arg^{445}-Ala-Gln-Arg/ Ser; yellow) and predicted subtilisin/kexin type 2 cleavage site in sea bass Amh (Arg^{426}-Ala-Thr-Arg/ Ala; red) are indicated. The light red box highlights conservation of the sea bass predicted cleavage site Arg^{426}-Ala-Thr-Arg among nine distantly related teleost species and gar. Amino acid similarities (80%) are boxed in grey.

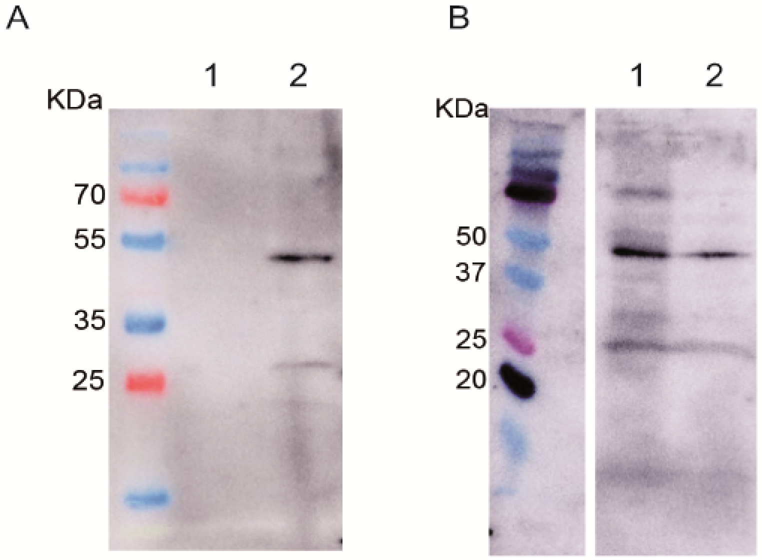

Figure S2. Specificity of the rabbit anti- sea bass Amhr2 antibody. (**A**) Total protein extracts from cells expressing the pcDNA3 empty vector as control (**A**, lane 1) and sea bass Amhr2 (**A**, lane 2). (**B**) Total protein extracts from sea bass previtellogenic ovary (**B**, lane 1) and from follicular cells (**B**, lane2). Approximately 40 μg of total protein from all extracts were analyzed. Protein standards (in kDa) were used to estimate protein molecular weight.

Table S1. Primers used in this study[a].

Primer	Position	Sequence (5′ → 3′) [b,c]		Annealing temp. [d]
*Bam*HI-amh1	29 [e]	ATAGGATCCCAGAGCAGGATGATGGTTGTG	sense	58 ºC → 68 ºC<br>70 ºC → 65 ºC
amh2	1460 [e]	ACTGTAAGGTGGTCAGCTGGA	antisense	64 ºC → 59 ºC
amh3	863 [e]	CTGAAGCGGTTCCTGGGCG	sense	52 ºC → 62 ºC
amh4-*Eco*RI	1704 [e]	ATCGAATTCACCAACTAAAGACTATGTGAAAC	antisense	58 ºC → 68 ºC<br>70 ºC → 65 ºC
amh10	1 [f,g]	AATGGGAATTCCTGCAGGTCTCACAAGGACT	sense	58 ºC → 68 ºC<br>65 ºC
amh12	1210 [g]	GAGAAGCGAGCTGACCCCAACAACCCA	sense	58 ºC → 68 ºC
amh13	1574 [f]	TACGACCTAGGTTTAGCGGCATCCACACTCC	antisense	58 ºC → 68 ºC<br>65 ºC
amh14	1247 [f]	gtggtggtgatggtggtgTCGCTTCTCCAGTCCTCTCTGCACCTCAT	antisense	58 ºC → 68 ºC
amh15	1210 [f]	GAGAAGCGAcaccaccatcaccaccacGCTGACCCCAACAACCCAG	sense	58 ºC → 68 ºC
amh16	1567 [g]	TACGACCTAGGTTAgtggtggtgatggtggtgACGACCTTCGATGCGGCATCCACACTCCCGTG	antisense	58 ºC → 68 ºC<br>65 ºC
amh17	1249 [g]	ACTGGGTTGTTGGGGTCAGCTCGCTTCTCCAGTCCTCTCTGCACCTCAT	antisense	58 ºC → 68 ºC
5′*AOX1*	855 [h]	GACTGGTTCCAATTGACAAGC	sense	54 ºC
3′ *AOX1*	1347 [h]	GGCAAATGGCATTCTGACATCCT	antisense	54 ºC

[a] Primers were obtained from Invitrogen or Isogen (Thermo Fisher Scientific; Isogen Life Science).
[b] Underlined sequences indicate restriction enzyme sites for cloning purposes. [c] Lower case letters code for Histidines.
[d] Touch-down or -up PCR maximum and minimum annealing temperatures.
[e] Position in *Dicentrarchus labrax amh* cDNA sequence with GenBank accession no. AM232701.1. [f] Position in *Dicentrarchus labrax amh* cDNA sequence in pPICK9-His6Amh.
[g] Position in *Dicentrarchus labrax amh* cDNA sequence pPICK9-AmhHis6. [h] Position in pPICK9 vector.

Table S2. Primers and hydrolysis probe used for quantitative real-time PCR.

Gene	Sequence (5′ → 3′) [a]	nM [d]	Amplicon size	Efficiency	First published
amhr2 (JQ801443.1) [b]	fw: CCATCCTGCGTTCTTGTTCA	300	67 bp	0.97	[A] ([30])
	rv: TGAGCAAGACCCATGTTTGC	300			
	pr: [6~FAM]AATCGCCACTGGTCGAGCCACAC[TAMRA]	125			
amh (AM232701.1) [b]	fw: TCCAAACACTGCTAACATCAACAA	50	74 bp	0.94	[B] ([21])
	rv: TGGCGTGGTTCTTGGGATT	300			
	pr: [6~FAM]CCATGGCTCATGTGCTTTCCCCCT[TAMRA]	125			
cyp19a1 (AJ311177.1) [b]	fw: TCCTCGCCGCTACTTCCA	300	65 bp	0.98	[C] ([61])
	rv: TGGCGATGTGCTTACCAACA	300			
	pr: [6 ~ FAM]CATTCGGTTCAGGCCCTCGCG[TAMRA]	100			
rpl13a (DT044539) [b]	fw: TCTGGAGGACTGTCAGGGGCATGC	100	148 bp	0.91	[D] ([72])
	rv: AGACGCACAATCTTGAGAGCAG	100			
luc1 (MH759210.1) [c]	fw: TACAACACCCCAACATCTTCGA	900	67 bp	0.96	This study
	rv: GGAAGTTCACCGGCGTCAT	900			
	pr: [6 ~ FAM]CGGGCGTGGCAGGTCTTCCC[TAMRA]	200			

[a] Forward (fw) and reverse (rv) primers were obtained from Invitrogen™ (Life Technologies). Hydrolysis probe (pr) was purchased from Eurofins Genomics, Germany.
[b] GenBank accession no. for sea bass gene.
[c] GenBank accession no. for *Photinus pyralis* voucher KSH 11044
[c] Amount of primer or probe in the PCR.

21. Halm, S.; Rocha, A.; Miura, T.; Prat, F.; Zanuy, S. Anti-Müllerian hormone (AMH/AMH) in the European sea bass: its gene structure, regulatory elements, and the expression of alternatively-spliced isoforms. *Gene* **2007**, *388*, 148–158, doi:10.1016/j.gene.2006.10.018.
30. Rocha, A.; Zanuy, S.; Gómez, A. Conserved anti-müllerian hormone: Anti-müllerian hormone type-2 receptor specific interaction and intracellular signaling in teleosts. *Biol. Reprod.* **2016**, *94*, doi:10.1095/biolreprod.115.137547.
61. Rocha, A.; Zanuy, S.; Carrillo, M.; Gómez, A. Seasonal changes in gonadal expression of gonadotropin receptors, steroidogenic acute regulatory protein and steroidogenic enzymes in the European sea bass. *Gen. Comp. Endocrinol.* **2009**, *162*, doi:10.1016/j.ygcen.2009.03.023.
72. Mitter, K.; Kotoulas, G.; Magoulas, A.; Mulero, V.; Sepulcre, P.; Figueras, A.; Novoa, B.; Sarropoulou, E. Evaluation of candidate reference genes for QPCR during ontogenesis and of immune-relevant tissues of European seabass (Dicentrarchus labrax). *Comp. Biochem. Physiol. Part B Biochem. Mol. Biol.* **2009**, *153*, 340–347, doi:https://doi.org/10.1016/j.cbpb.2009.04.009.
